# Determinants of infant feeding practices among Black mothers living with HIV: a multinomial logistic regression analysis

**DOI:** 10.1186/s12889-021-10675-2

**Published:** 2021-04-07

**Authors:** Josephine Etowa, Jean Hannan, Egbe B. Etowa, Seye Babatunde, J. Craig Phillips

**Affiliations:** 1 School of Nursing, Faculty of Health Sciences, University of Ottawa, 451 Smyth Road, Ottawa, Ontario K1H 8M5 Canada; 2 Nicole Wertheim College of Nursing and Health Sciences, Florida International University, 11200 Southwest 8th Street, Miami, FL 33199 USA; 3 Department of Sociology, Anthropology & Criminology; Faculty of Arts, Humanities & Social Sciences, 401 Sunset Avenue, Windsor, Ontario N9B 3P4 Canada; 4 Centre for Health and Development, University of Port Harcourt, Port Harcourt, Nigeria

**Keywords:** Infant feeding, HIV, Black mothers, Sociocultural and psychosocial determinants, Healthcare

## Abstract

**Background:**

Infant feeding practices are imperative for babies’ and mothers’ health and emotional wellbeing. Although infant feeding may seem simple, the decisions surrounding it are complex and have far-reaching implications for women globally. This is an especially difficult concern among mothers living with HIV because breastfeeding can transmit HIV from mother to child. This is further complicated by cultural expectations in case of Black mothers living with HIV. This paper discusses determinants of infant feeding practices among Black mothers living with HIV who were on anti-retroviral therapy (ART) in two North American cites and one African city.

**Methods:**

A cross-sectional, multi-country survey using venue-based convenience sampling of Black mothers living with HIV was employed. The effective response rates were 89% (*n* = 89) in Ottawa, Canada; 67% (*n* = 201) in Miami, Florida, US; and 100% (*n* = 400) in Port Harcourt, Nigeria, equaling a total sample size of 690. Data were collected in Qualtrics and managed in Excel and SPSS. Multinomial logistic regression analyses were used to determine the factors influencing the mothers’ infant feeding practices (Exclusive Formula Feeding [EFF] = 1; Mixed Feeding [MF] = 2; and Exclusive Breastfeeding [EBF while on ART] =3).

**Results:**

The results highlight socio-demographics, EFF determinants**,** and EBF determinants**.** The statistically significant determinants of infant feeding practices included national guideline on infant feeding, cultural beliefs and practices, healthcare systems, healthcare personnel, infant feeding attitudes, social support, and perceived stress. Mothers’ mean ages were Ottawa (36.6 ± 6.4), Miami (32.4 ± 5.8), and Port Harcourt (34.7 ± 5.7). All sampled women gave birth to least one infant after their HIV diagnoses. Statistically significant (*p* < .05) determinants of EFF relative to MF were the national guideline of EFF (relative risk [RR] = 218.19), cultural beliefs (RR = .15), received healthcare (RR = 21.17), received healthcare through a nurse/midwife (RR = 3.1), and perceived stress (RR = .9). Statistically significant determinants of EBF relative to MF were received healthcare (RR = 20.26), received healthcare through a nurse/midwife (RR = 2.31), functional social support (RR = 1.07), and perceived stress (RR = .9).

**Conclusion:**

While cultural beliefs and perceived stress favoured MF over EFF, advice of healthcare workers, and the care received from a nurse/midwife improved EFF over MF. Also while the mothers’ perceived stress favoured MF over EBF, advice of their nurses or midwife and the social support improved EBF over MF. The providers advice was congruent with WHO and national guidelines for infant feeding among mothers living with HIV. These results have implications for nursing, healthcare practice, and policies on infant feeding practices for mothers living with HIV.

## Background

Infant feeding practices are imperative for babies’ and mothers’ health and emotional wellbeing. Although infant feeding may seem simple, the decisions surrounding it are complex and have far-reaching implications for women globally. This is especially difficult for mothers living with HIV because breastfeeding can transmit HIV from mother to child [1]. Thus, eliminating mother to child transmission (MTCT) of HIV is a global health priority in the fight against HIV and in breaking the vicious cycle of HIV transmission to newborns [2–4].

In 2010, the World Health Organization (WHO) recommended that mothers living with HIV adopt Exclusive Breastfeeding (EBF) for up to 6 months and continue breastfeeding until the infant was 12 months old, provided that they and/or the infants were taking antiretroviral therapy (ART) during the same period [5]. The 2016 update to this recommendation states that, as long as the mother is on lifelong ART, she can continue breastfeeding for up to 24 months or longer until a nutritionally adequate and safe diet without breast milk can be provided [6]. However, the 2016 update did not replace the 2010 guidelines, and mothers living with HIV are recommended to avoid breastfeeding if formula feeding is accessible, feasible, affordable, sustainable, and safe. Consequently, the implementation of these guidelines varies globally [4]. High income countries, such as Canada and the United States (US) recommend EFF, while low- to middle-income countries, such as Nigeria, recommend EBF while on ART [7]. Although being on ART and monitoring viral are a critical factor, some mothers in Nigeria reported that they would still breastfeed their children, irrespective of their HIV status [8]. In southwestern Nigeria, breastfeeding is perceived as essential to a baby’s health – strengthening the physical and spiritual bonds between mothers and children [9]. In contrast, women in Canada and the US risk prosecution when potentially transmitting HIV to infants through breastfeeding [10]. Even with a less than 2% aggregated population risk of vertical transmission when the mother is on ART [11], the potential for prosecution remains in both countries [12].

HIV remains an important public health concern in Canada and the US, where achievements in reducing the MTCT of HIV, which is an intense stress factor for many women living with HIV, has arisen from offering HIV testing and providing ART to pregnant women [13]. Aside from the tension emanating from the differing guideline implementation between countries of residence and origin, immigrant Black women, especially those living in high income countries, face a more intense dilemma of either yielding to existing cultural norms on mothering [5] or adhering to public health policy on infant feeding. Culture influences the desire for a particular style of mothering, whether the mother is living with HIV or not [14]. Hence, infant feeding practices among mothers living with HIV must be understood via their sociocultural contexts, with due consideration given to the mothers’ mental health conditions accentuated by HIV.

Studies among African immigrant women have shown that they upheld their cultural beliefs about infant feeding upon moving to Canada and that the desire to breastfeed was a common existential perception shared by African women, including those living with HIV [15–17]. At onset of immigration to the North America, Black mothers bring in the culture of breastfeeding and are more likely to breastfeed, but with time Formula feeding acculturation sets in [18, 19], especially with changes in socioeconomic status and occupational demands. In the 1970s at the boom of formula industries, many Black mothers would have already imbibed the culture of formula feeding before migration to North America, and until recently a few well-educated and in the working class would hold the culture of formula feeding prior to migration [18, 20]. In contrast, among American born Black mothers breastfeeding culture is more common among women who are older, married, and possess high levels of education, even though breastfeeding rates is generally lower in African American mothers than other ethnic groups in the US [21]. Hence cultural variations in infant feeding practices may exists between migrants versus non-migrant Black mothers, and between Black mothers of different socioeconomic status.

A study of Black mothers in Canada documented that adherence to the national guidelines of adopting EFF while living with HIV was associated with difficulty in maintaining their ascribed social roles as respected wives, mothers, and members of society [3]. Additionally, the fear of alienation and stigmatization often makes it difficult for women to decide not to breastfeed [22]. This tension associated with adherence to national guidelines and meeting cultural expectations is a source of significant stress for mothers, who feel pressure from both society and themselves [23, 24]. Health providers in a US study reported non-adherence to EFF by their HIV+ patients due to the stigma associated with not breastfeeding [25]. Thus, Black women living with HIV in Canada and the US may be caught between conflicting cultural expectations, public policies, and legal obligations related to infant feeding. However, no studies have specifically examined the infant feeding practices and the sociocultural context of motherhood among Black women, especially those living with HIV in high income countries.

Although this paper primarily presents quantitative results, this has been drawn from a broader mixed-methods study, which sought to understand the experiences of Black mothers residing in two cities in two high income countries (Ottawa, Canada, and Miami, Florida, US) *vis a vis* those of indigenous Black women in Port Harcourt, Nigeria [26]. This paper has identified the determinants of infant feeding practices among Black mothers living with HIV in the two North American cities compared to those in the African city of Port Harcourt. Black mothers in this context were African, Caribbean, and Black (ACB) mothers residing in Ottawa, Canada; Miami-Florida, USA; or Port Harcourt, Nigeria. It includes women of African descent who were either born in these cities or those who migrated into those cities from elsewhere. Analysis of the different infant feeding practices of the Black mothers living with HIV in these three sites may inform health care education, practice, and policy.

## Methods

This research employed a cross-sectional, multi-country survey using venue-based convenience sampling of Black mothers living with HIV. Venue-based sampling was employed because probability sampling was highly inefficient due to several factors including the relatively small size of ACB population in both Ottawa, Canada and Miami, US. In Canada ACB people make up only 3.5% of the Canada’s total population [27], and this in part explain the small sample size of Ottawa site. Moreover, persons living with HIV are a hard to reach population, and reaching Black women proved to be even harder among people living with HIV who had a baby after HIV diagnosis. Only Black women who had babies since 2010 when the first WHO infant feeding guidelines were developed were recruited. The implementation of WHO guideline in 2010 marked a global milestone in HIV care for this population. The sensitivity of the topic, including fear of self-disclosing HIV status and stigma were also obstacles to recruitment by probability sampling. Sampling were done at all identified event venues of the mothers to reduce sampling error. The venues were identified community resource centers, public health facilities and AIDS service organizations where people living with HIV will normally meet or receive health care. Honorarium were given to participants in the study. Trained research assistants, graduate students and project staff recruited participants from the identified venues between 2016 and 2018. Data collection was initiated in 2016 and it continued until 2018 because of the difficulty of reaching participants in a shorter period. Data collection instruments included paper or electronic (online or offline) survey questionnaire using a computer tablet, depending on participants’ preference. For confidentiality and anonymity, all participant data were coded using study-generated identification entered in Qualtrics ^XM^ [28]. The data were later downloaded and managed in Excel and SPSS files.

We estimated a minimum sample sizes of 800 from all three sites. This was large enough to detect small to moderate effects observed between variables in quantitative analysis assuming an alpha of .05 and power of .8. Hence based on the relative frequencies of Black, HIV-positive women of child-bearing age making an infant feeding choice at desired precision level of 5%, estimated sample size in each site were: Ottawa (*n* = 100); Port Harcourt (*n* = 400); and Miami (*n* = 300). The effective response rates were: 89% (*n* = 89) in Ottawa, 67% (*n* = 201) in Miami, and 100% (*n* = 400) in Port Harcourt, giving a total sample size of 690. The effective response rates were determined by the ratio of surveys questionnaire completed to the total number of survey questionnaire issued per site multiplied by 100. The inclusion criteria were as follows: being an African, a Caribbean, or Black mother who migrated or was born in Canada; living with HIV; and having given birth to at least one child after HIV diagnosis. Almost all the mothers were on ART. All participants were between 18 and 49 years of age and resided in one of the three study sites. Participants completed questionnaires to provide demographic information, infant feeding practices, sociocultural characteristics, prenatal information, and healthcare information. They also completed instruments assessing their psychosocial attributes, including functional social support, infant feeding attitudes and perceived stress.

The Functional Social support scale was used, and this has been described in a previous study [29]. It is a 7-items scale adapted from the Duke-UNC Functional Social Support Questionnaire [30], Cohen perceived stress scale and Iowa Infant Feeding Attitude Scales were used as described in previous work, Infant feeding attitudes was measured using the 17-item validated Iowa Infant Feeding Attitude Scale (IIFAS) [31]. A maximum score of 85 was attainable on the scale with high score showing positive attitude towards breastfeeding. Stress was rated based on 10-Items Cohen Perceived Stress Scale [32, 33] . The Scale (PSS) scale is validated and have been used among diverse populations including among mothers living with HIV. A total score of 40 was attainable on the scale. Higher scores show higher levels of stress.

Multinomial logistic regression analyses were used to estimate determinants of the mothers’ infant feeding practices. These were conducted in five consecutive levels of SPSS analyses, designated as Models 1 to 5 (Tables 5 and 6). Each block of selected determinants, added to the consecutive model at each level of analysis significantly improved the models’ fits, and the final level, Model 5, was chosen to explain the relationship. Sociodemographic variables (e.g., age, education) were controlled for in this model. The levels of statistical significance were set at an alpha criterion of *p* < .05. Infant feeding practices, the outcome (dependent) variable in these analyses was categorical with three outcome levels (EFF = 1, MF = 2 and EBF = 3). MF was used as the reference category because it signified non-adherence to infant feeding guidelines in all national contexts. Blocks of predictor (independent) variables were selected based on pre-defined factors in research protocols informed by a systematic review preceding this work [26]. Explicit conceptual framework on variables selection is provided in the research protocol [34]. Details of these blocks of independent variables were entered into each of the successive multinomial logistic regression models, and their measurements are described below.

In Model 1, “national policy guideline” was the only included variable. It was dichotomized as guideline of EFF =1, and guideline of EBF while on ART = 0. National guideline on infant feeding, a priori, was expected to be a major determinant of infant feeding practices. Therefore, it was included alone in Model 1 to study and separate its unique effect before inclusion of other variables in subsequent models. In Model 2, socio-demographic variables were entered: mother’s age in years, marital status (married = 1, not married = 0), number of children born after HIV diagnosis, number of years of formal education, and type of employment (salaried or waged employment = 1, otherwise = 0). This block of variables have statistically significant (*p* < .05) contributory effect on Model 2, justifying its inclusion in subsequent models. In Model 3, sociocultural factors were included. The sociocultural factors were spouse or baby’s father’s influence, family influence, cultural beliefs, healthcare, healthcare workers. To measure these sociocultural factors, we transformed their respective scoring on the Influence of Specific Referent Scale (ISR) [35] to binary variables. For example, mothers were asked: “How important is your baby’s father’s opinion to you about how to feed your baby?” The ISR Scale options of “very unimportant,” “unimportant,” “unsure,” “important” and “very important” were coded as 1 for a response of “important” or “very important” (proxy = important) and 0 for all other values (proxy = unimportant). In Model 4, healthcare-related variables were entered. Mothers were asked: “Did you receive healthcare during the period you were pregnant and living with HIV?”. If the response was “yes,” the answer was coded as 1, and “no” was coded as 0. Mothers were then asked from whom they received prenatal care. A response of “nurse/midwife” was coded as 1, and a response of “medical doctor/clinical officer” was coded as 0. Responses to other categories for this question on sources of prenatal care included “service providers” (*n* = 2), “I choose not to answer” (*n* = 8) and “missing values” (*n* = 9). All these were excluded by listwise deletion from the analyses. Deleted observations were not uniquely different from undeleted observations and account for 11.3% (*n* = 85) of the total sample size (*N* = 690). The final model, Model 5, incorporated variables from previously validated psychosocial instruments expected to influence infant feeding practices. Functional social support, Cohen’s Perceived Stress Scale (PSS), and the Iowa Infant Feeding Attitude Scale (IIFAS) were entered as continuous variables, in addition to the previous determinants already in the model.

## Results

This section presents three subsections of the findings: 1) sociodemographic characteristics of the study population; 2) determinants of EFF relative to MF; and 3) determinants of EBF relative to MF

### Sociodemographic characteristics of the study population

A preliminary description of sociodemographic variables was necessary because their distribution is a well-known potential source of variability among many outcome variables, including variations in infant feeding practices in mothers living with HIV [36–38]. Hence, Table 1 summarizes the key demographic characteristics of the ACB mothers living with HIV.

**Table 1:** Socioeconomic characteristics of the mothers

Socioeconomic characteristics	Ottawa, Canada n (%)	Port Harcourt, Nigeria n (%)	Miami-Florida, USA n (%)	All Sites n (%)
Mothers’ age (categories)				
18–24 years	2 (2.9)	18 (4.5)	15 (8.3)	35 (5.4))
25–34 years	20 (29.4)	180 (45.0)	91 (50.3)	291 (44.8)
35–44 years	37 (54.4)	184 (46.0)	74 (40.9)	295 (45.5)
45–49 years	9 (13.2)	18 (4.5)	1 (0.5)	28 (4.3)
Total valid responses	68 (100)	400 (100)	181 (100)	649 (100)
Relationship status:				
Single/separated/divorced/widowed	57 (66.3)	57 (14.4)	61 (33.5)	175 (26.3)
Married	29 (33.7)	340 (85.6)	121 (66.5)	490 (73.7)
Total valid responses	86 (100)	397 (100)	182 (100)	665 (100)
Number of persons in household				
< 5 persons	56 (66.7)	222 (56.1)	125 (77.2)	403 (62.8)
≥ 5 persons	28 (33.3)	174 (43.9)	37 (22.8)	239 (37.2)
Total valid responses	84 (100)	396 (100)	162 (100)	642 (100)
Number of children born after HIV+				
< 3 children	43 (87.8)	372 (93.0)	193 (96.0)	608 (93.5)
≥ 3 children	6 (12.2)	28 (7.0)	8 (4.0)	42 (6.5)
Total valid responses	49 (100)	400 (100)	201 (100)	650 (100)
Number of years since HIV+				
1–5 years	9 (12.2)	178 (45.2)	29 (24.4)	216 (36.8))
6–10 years	24 (32.4)	166 (42.1)	28 (23.5)	218 (37.1)
> 10 years	41 (55.4)	50 (12.7)	62 (52.1)	153 (26.1)
Total valid responses	74 (100)	394 (100)	119 (100)	587 (100)
On HIV treatment (ART)?				
Yes	50 (96.2)	399 (99.8)	187 (97.4)	636 (98.8)
No	0 (0.0)	1 (0.2)	0 (0.0)	1 (0.1)
I choose not to answer	2 (3.8)	0 (0.0)	5 (2.6)	7 (1.1)
Total valid responses	52 (100)	400 (100)	192 (100)	644 (100)
Education:				
Primary school	1 (1.2)	42 (10.7)	0 (0.0)	43 (6.4)
High school, technical or vocational school	34 (40.0)	250 (63.4)	131 (66.5)	415 (61.4)
College or university	50 (58.8)	102 (25.9)	66 (33.5)	218 (32.2)
Total valid responses	85 (100)	394 (100)	187 (100)	676 (100)
Employment status:				
Employed (full time or part time)	51 (57.3)	320 (87.9)	65 (32.7)	436 (66.9)
Unemployed	38 (42.7)	44 (12.1)	134 (67.3)	216 (33.1)
Total valid responses	89 (100)	364 (100)	189 (100)	652 (100)
Main source of income:				
Wages or salaries	40 (48.8)	66 (20.1)	64 (40.3)	170 (29.9)
Self employment income	5 (6.1)	260 (79.3)	8 (5.0)	273 (48.0)
Social assistance	20 (24.4)	0 (0.0)	77 (48.4)	97 (17.0)
Other income sources	17 (20.7)	2 (0.6)	10 (6.3)	29 (5.1)
Total valid responses	82 (100)	328 (100)	159 (100)	569 (100)

Average age of the mothers was 34 years, however, the largest percentage of them were aged 35–44 years in Ottawa (*n* = 37, 54.4%). In Port Harcourt, almost equal majority of the women were aged 25–34 years (*n* = 180, 45.0%) and 3–44 years (*n* = 184, 46.0%). The largest percentage of the women were aged 25 to 34 years in Miami (*n* = 91, 50.3%). Most mothers were either single, separated, divorced or widowed in Ottawa (*n* = 57, 66.3%) and the greater percentage were married in Port Harcourt (*n* = 340, 85.6%) and in Miami (*n* = 121, 66.5%). Household sizes were mostly small (less than 5 persons) in Ottawa (*n* = 55, 66.7%), Port Harcourt (*n* = 222, 56.1%) and Miami (*n* = 125, 77.2%). All mothers had at least a baby after being diagnosed of HIV, and almost all gave birth to only one or two children since living with HIV in Ottawa (*n* = 43, 87.8%), Port Harcourt (*n* = 372, 93.0%), and Miami (*n* = 193, 96.0%). The average number of years since HIV diagnosis was 8.1 years with the majority diagnosed for HIV since more than 10 years ago in Ottawa (*n* = 41, 55.4%), and Miami (*n* = 62, 52.1%). Most mothers in Port Harcourt were either diagnosed in the past 5 years (*n* = 178, 45.2%) or 6 to 10 years (*n* = 166, 42.1%) prior to the survey. Almost all mothers were on ART in Ottawa (*n* = 50, 96.2%), Port Harcourt (*n* = 399, 99.8%) and Miami (*n* = 187, 97.4%). Most of the mothers in Port Harcourt had completed high school, technical or vocational education (*n* = 415, 61.4%), or completed a college or a university education (*n* = 218, 32.2%). Miami had largest percentage (*n* = 131, 66.5%) of mothers who completed high school, technical or vocational education while 33.5% (*n* = 66) or completed a college or a university education (*n* = 66, 33.5%). In Port Harcourt, 63.4% (*n* = 210) completed high school, technical or vocational education while 25.9% (*n* = 103) finished a college or a university education. Mothers reported a wide range of employment (full- or part-time), with the highest rate in Port Harcourt (*n* = 320, 87.9%) and the lowest in Miami (*n* = 65, 32.7%).

Table 2 provides descriptive statistics for the mothers’ infant feeding practices. It also describes each mother’s perception of her social network’s opinions about appropriate infant feeding methods. Most Black mothers living with HIV practiced EFF in Ottawa (*n* = 79, 90.8%) and Miami (*n* = 146, 75.7%). In contrast, only 57 (18.1%) mothers in Port Harcourt practiced EFF; most of them (*n* = 210, 66.7%) practiced EBF by following their national guidelines.

**Table 2:** Sociocultural variables related to infant feeding practices of the Black mothers living with HIV

Indicator	Ottawa, Canada n (%)	Port Harcourt, Nigeria n (%)	Miami-Florida, USA n (%)	All Sites n (%)
When it comes to feeding your baby, do you practice exclusive formula feeding?				
Yes, exclusive formula feeding	79 (90.8)	57 (18.1)	146 (75.6)	282 (47.4)
No, mixed feeding	3 (3.4)	48 (15.2)	14 (7.3)	65 (10.9)
No, exclusive breastfeeding	5 (5.8)	210 (66.7)	10 (5.2)	225 (37.8)
I choose not to answer	0 (0.0)	0 (0.0)	23 (11.9)	23 (3.9)
Total valid responses	87 (100)	315 (100)	193 (100)	595 (100)
Your spouse/partner/baby’s father thinks you should practice?				
Exclusive formula feeding	56 (65.1)	57 (17.9)	106 (53.0)	219 (36.2)
Mix feed	4 (4.7)	49 (15.3)	10 (5.0)	63 (10.4)
Exclusive breastfeeding	10 (11.6)	213 (66.8)	20 (10.0)	243 (40.2)
I choose not to answer	16 (18.6)	0 (0.0)	64 (32.0)	80 (13.2)
Total valid responses	86 (100)	319 (100)	200 (100)	605 (100)
Your other family members or close relatives thinks you should practice?				
Exclusive formula feeding	13 (15.3)	35 (12.0)	114 (56.7)	162 (28.1)
Mix feed	7 (8.2)	86 (29.7)	14 (7.0)	107 (18.6)
Exclusive breastfeeding	57 (67.1)	114 (39.3)	25 (12.4)	196 (34.0)
I choose not to answer	8 (9.4)	55 (19.0)	48 (23.9)	111 (19.3)
Total valid responses	85 (100)	290 (100)	201 (100)	576 (100)
What type infant feeding do your healthcare provider think you should practice?				
Exclusive formula feeding	78 (89.7)	37 (11.9)	162 (80.6)	277 (46.3)
Mix feed	4 (4.6	2 (0.6)	6 (3.0)	12 (2.0)
Exclusive breastfeeding	4 (4.6)	269 (86.7)	14 (7.0)	287 (48.0)
I choose not to answer	1 (1.1)	2 (0.6)	19 (9.4)	22 (3.7)
Total valid responses	87 (100)	310 (100)	201 (100)	598 (100)
Are there cultural beliefs and practices in your place of origin about methods of feeding your baby?				
Yes	59 (67.8)	26 (6.6)	62 (30.8)	147 (21.5)
No	17 (19.6)	367 (92.9)	88 (43.8)	472 (69.1)
I choose not to answer	11 (12.6)	2 (0.5)	51 (25.4)	64 (9.4)
Total valid responses	87 (100)	395 (100)	201 (100)	683 (100)
Does the cultural beliefs oppose any of the infant feeding methods?				
Yes, exclusive breast feeding	18 (22.5)	8 (12.7)	27 (14.0)	53 (15.8)
Yes, mix feeding	7 (8.8)	4 (6.4)	13 (6.7)	24 (7.1)
Yes, exclusive formula feeding	29 (36.2)	12 (19.0)	59 (30.6)	100 (29.8)
I choose not to answer	26 (32.5)	39 (61.9)	94 (48.7)	159 (47.3)
Total valid responses	80 (100)	63 (100)	193 (100)	336 (100)

Most of the mothers in Ottawa (*n* = 56, 65.1%) and Miami (*n* = 106, 53.0%) perceived that their spouses, partners or baby’s fathers supported EFF. In Ottawa, most (*n* = 57, 67.1%) other family members (grandparents, siblings, in-laws, etc.) supported EBF, while the majority (*n* = 114, 56.7%) supported EFF in Miami. In Port Harcourt, most (*n* = 213, 66.8%) of the women reported that their spouses, partners or baby’s fathers supported EBF. Proportions of the mothers in Port Harcourt who reported other relatives’ opinions about infant feeding practices included EFF (*n* = 35, 12.1%); MF (*n* = 86, 29.7%) and EBF (*n* = 114, 39.3%). In Port Harcourt, 55 mothers (19.0%) chose not to answer to the question of other relatives’ opinion on their infant feeding practices. Most mothers in Ottawa (*n* = 78, 89.7%) and Miami (*n* = 162, 80.6%) perceived that their health providers supported EFF, and, in Port Harcourt (*n* = 269, 84.3%), most of them believed their health providers promoted EBF. Mothers had divergent views about whether their cultural beliefs or practices inhibited infant feeding practices. In Ottawa, 18 mothers (22.5%) reported that their culture of origin prohibited EFF; seven (8.8%) believed their culture disallowed MF; 29 (36.3%) explained that their culture forbade EBF; and 26 (32.5%) chose not to answer. Similarly, in Miami, 27 mothers (14.0%) reported that their culture of origin culture prohibited EFF; 13 (6.7%) believed their culture disallowed MF; 59 (30.6%) stated that their culture forbade EBF; and 94 (48.7%) chose not to answer. In Port Harcourt, responses to this question about the influence of culture were sparse, with only 63 valid responses out of 400 respondents. The greater percent of the mothers did not answer this question. Based on the valid responses, eight mothers (12.7%) reported that their culture of origin prohibited EFF, four (6.3%) viewed their culture as disallowing MF, 12 (19.0%) believed their culture forbade EBF; and 39 (61.9%) chose not to answer.

Table 3 describes statistics of other sociocultural variables, some of which were included in the analysis model. These variables included healthcare information during pregnancy while living with HIV, mothers’ ratings of their spouses, and other family members’ opinions about infant feeding practices, mothers’ ratings of health providers’ views about infant feeding options, and mothers’ ratings of how cultural beliefs and practices influence their methods of feeding their babies. Concerning healthcare information, the mothers were asked if they received health care during pregnancy while living with HIV, they were also asked to name the category of health care staff that rendered the health care services. Almost all mothers from all sites (*n* = 659, 96.6%) received health care during their pregnancy while living with HIV. In Ottawa (*n* = 85, 96.6%), Miami (*n* = 179, 91.3%) and Port Harcourt (*n* = 395, 99.2%) – received healthcare from healthcare personnel when they were pregnant while living with HIV. A larger percentage of the mothers received the health care from medical doctor or a clinical officer in Ottawa (*n* = 47, 55.3%) and in Miami (*n* = 151, 84.4). In contrast, most of the mothers in in Port Harcourt (*n* = 305, 77.2%) received health care from a nurse.

**Table 3:** Other sociocultural factors that potentially influence infant feeding practices

Indicator	Ottawa, Canada n (%)	Port Harcourt, Nigeria n (%)	Miami-Florida, USA n (%)	All Sites n (%)
Did you receive health care during pregnancy?				
Yes	85 (96.6)	395 (99.2)	179 (91.3)	659 (96.6)
No	3 (3.4)	3 (0.8)	17 (8.7)	23 (3.4)
Total valid responses	88 (100)	398 (100)	196 (100)	684 (100)
From whom did you receive health care during pregnancy?				
Medical doctor, clinical officer	47 (55.3)	88 (22.3)	151 (84.4)	286 (43.4)
Nurse, Midwife	6 (7.1)	305 (77.2)	7 (3.9)	318 (48.2)
Other sources	32 (37.6)	2 (0.5)	21 (11.7)	55 (9.4)
Total valid responses	85 (100)	395 (100)	179 (100)	659 (100)
How important is your spouse/partner/baby’s father’s opinion to you about how to feed your baby?				
Very unimportant, unimportant, unsure	20 (23.0)	72 (18.8)	65 (32.3)	157 (23.0)
Important, very important	62 (71.3)	310 (78.3)	108 (53.7)	480 (70.2)
I choose not to answer	5 (5.7)	14 (3.5)	28 (14.0)	47 (6.8)
Total valid responses	87 (100)	396 (100)	201 (100)	684 (100)
How much do you care about family members opinion about feeding your baby?				
Do not care at all, do not care, unsure	20 (22.5)	194 (52.4)	77 (38.3)	291 (44.1)
Care, care very much	66 (74.1)	168 (45.4)	97 (48.3)	331 (50.1)
I choose not to answer	3 (3.4)	8 (2.2)	27 (13.4)	38 (5.8)
Total valid responses	89 (100)	370 (100)	201 (100)	660 (100)
How much do you care about your healthcare provider’s opinion about feeding your baby?				
Do not care at all, do not care or unsure	0 (0.0)	4 (1.0)	12 (6.0)	16 (2.3)
Care or care very much	89 (100)	393 (98.5)	170 (84.6)	652 (94.6)
I choose not to answer	0 (0.0)	2 (0.5)	19 (9.4)	21 (3.1)
Total valid responses	89 (100)	399 (100)	201 (100)	689 (100)
How much do your cultural beliefs/practices influence your infant feeding choices?				
Not at all, Not much, unsure	44 (49.4)	383 (96.2)	70 (47.9)	497 (78.5)
Much or very much	43 (48.3)	13 (3.3.)	60 (41.1)	116 (18.3)
I choose not to answer	2 (2.3)	2 (0.5)	16 (11.0)	20 (3.2)
Total valid responses	89 (100)	398 (100)	146 (100)	633 (100)

Most mothers in all sites (*n* = 480, 70.2%) rated the opinions of their spouses/partners/babies’ fathers about infant feeding as being “important” or “very important.” At individual site levels, this rating was broken down into Ottawa (*n* = 62, 71.3%), Miami (*n* = 108, 53.7%) and Port Harcourt (*n* = 310, 78.3%). Most mothers reported that they either “cared” or “cared very much” about other family members’ (grandmothers, mothers-in-law, siblings, etc.) opinions about infant feeding practices in Ottawa (*n* = 66, 74.1%) and Miami (*n* = 97, 48.3%). In Port Harcourt, however, the majority (*n* = 194, 53.6%) did not care at all, did not care or were unsure about their other family members’ opinions on infant feeding methods. Mothers rated healthcare providers exceptionally highly, as all women in Ottawa (*n* = 89, 100%) either “cared” or “cared very much” about their healthcare providers’ recommendations. Also, almost all mothers in Port Harcourt (*n* = 393, 98.5%) and Miami (*n* = 170, 84.6%) either “cared” or “cared very much” about their healthcare providers’ infant feeding recommendations. When asked how their cultural beliefs and practices influenced their infant feeding practices, most mothers in all three sites (*n* = 497, 78.5%) reported either “not at all,” “not much” or “unsure.” Site specific breakdown show that about half of the mothers who provided valid responses in Ottawa (*n* = 43, 49.4%) and Miami (*n* = 60, 46.1%) rated the influence of cultural beliefs and practices on their infant feeding practices as “not at all,” “not much,” or “unsure.” In Port Harcourt, the vast majority (*n* = 393, 96.2%) felt that the reported the influence of cultural beliefs and practices on their infant feeding practices as “not at all,” “not much” or “unsure.”

Table 4 provides mean scale scores for the three psychosocial parameters included in the multinomial logistic regression. Mean scores on the Iowa infant feeding attitudes scale were Ottawa (59.1 ± 7.0), Miami (52.6 ± 7.0) and Port Harcourt (55.7 ± 6.9) out of a total of 85. Mean functional social support scores were Ottawa (24.8 ± 6.6), Miami (21.9 ± 8.1) and Port Harcourt (20.7 ± 6.2) out of a total of 35. Mean scores on Cohen’s perceived stress scale were Ottawa (15.2 ± 6.5), Miami (21.1 ± 6.9) and Port Harcourt (22.1 ± 4.2) out of a total of 40.

**Table 4:** Psychosocial variables of the Black mothers included in the multinomial logistic model

Characteristics	Ottawa, Canada (M ± SD)	Port Harcourt, Nigeria (M ± SD)	Miami-Florida, USA (M ± SD)	Overall (M ± SD)	Maximum score attainable
Iowa infant feeding attitude score	59.1 ± 7.0	55.72 ± 6.1	52.6 ± 6.9	55.2 ± 6.9	85
Functional social support score	24.8 ± 6.6	20.7 ± 6.2	21.9 ± 8.1	21.6 ± 7.0	35
Cohen’s perceived stress score	15.2 ± 6.5	21.1 ± 4.7	21.1 ± 6.9	20.7 ± 5.9	40

The result of the multinomial logistic analysis is split in Tables 5 and 6 to show parameter estimates of EFF versus MF and EBF versus MF, respectively. Model in each of the tables is the final step of analysis, containing comprehensive entries of predictor variables, which yielded a statistically significant fitted model (*X*^*2*^ = 243.3, *p* < 0.05) with a relatively reduced error estimate (− 2 log-likelihood = 485.2) and an accuracy of 75.1%. Based on their significant (*X*^*2*^) estimates, each of the consecutive models (1 to 4) in Tables 5 and 6 show that each step in the analysis had a significant (*p* < 0.05) contribution to Model 5.

**Table 5:** Results of multinomial logistic regression to determine factors of infant feeding practices (EFF versus MF)

Effects	Model 1	Model 2	Model 3	Model 4	Model 5
National Guideline (EFF = 1, EBF + ART =0)	13.6* (0.3)	18.3* (0.4)	35.4* (0.6)	76.7* (0.8)	218.2* (0.9)
Age (Years)		1.1*(< 0.1)	1.4 (< 0.1)	1.1 (< 0.1)	1.1 (< 0.1)
Marital Status (Married =1, Otherwise = 0)		0.7 (0.4)	0.6 (0.5)	0.7 (0.5)	0.8 (0.5)
Children born after diagnosed HIV Positive (headcount)		0.9 (0.2)	1.0 (0.3)	0.9 (0.3)	0.8 (0.3)
Formal education (Years)		0.9 (0.1)	0.9 (0.1)	0.8* (0.1)	0.9 (0.1)
Employment (Salaried/waged =1, otherwise =0)		1.2 (0.4)	1.0 (0.5)	0.9 (0.5)	0.8 (0.6)
Rating of Baby’s father’s opinion about infant feeding (Important =1, Otherwise =0)			0.8 (0.5)	0.6 (0.5)	0.7 (0.5)
Concerns about family members’ opinion on infant feeding (cared =1, otherwise =0)			0.9 (0.4)	0.7 (0.4)	0.8 (0.4)
Are there cultural beliefs/ traditions in your place of origin about methods of feeding your baby? (Yes = 1, No = 0)			0.2* (0.6)	0.2* (0.6)	0.2* (0.7)
Received health care during pregnancy? (yes = 1, No =0)				14.8* (0.9)	21.2* (1.1)
Received healthcare from? (Nurse =1, Medical doctor/clinical officer =0)				2.6 (1.0)	3.1* (0.5)
Infant feeding attitude (score on Iowa scale)					1.1 (< 0.1)
Functional Social Support (score on scale)					1.04 (< 0.1)
Perceived Stress (score on Cohen’s scale)					0.9* (0.1)
*Model summary*					
(−2 Log Likelihood)	20.2	543.0	532.5	490.5	485.2
Chi-square statistics (*X*^*2*^)	339.4*	312.3*	231.6*	229.2*	243.3*
Observations analysed, n (%)	373 (54.1)				
Accuracy (%)	76.8	75.3	73.1	74.6	75.1

***p < 0.001, **p < 0.01, *p < 0.05

**Table 6:** Results of multinomial logistic regression to determine factors of infant feeding practices (EBF versus MF)

Effects	Model 1	Model 2	Model 3	Model 4	Model 5
National guideline (EFF =1, EBF + ART =0)	0.2* (0.4)	0.2* (0.5)	0.3 (0.6)	0.5 (0.7)	0.7 (0.9)
Age (Years)		1.1*(< 0.1)	1.04 (< 0.1)	1.1 (< 0.1)	1.0 (< 0.1)
Marital Status (Married =1, Otherwise = 0)		1.2 (0.4)	1.1 (0.5)	1.2 (0.5)	1.4 (0.5)
Children born after diagnosed HIV Positive (headcount)		0.6 (0.2)	0.7 (0.2)	0.7 (0.2)	0.7 (0.3)
Formal education (Years)		1.0 (0.1)	1.0 (0.1)	1.0 (0.1)	1.0 (< 0.1)
Employment (Salaried/waged =1, otherwise =0)		2.2 (0.4)	1.7 (0.4)	1.9 (0.5)	2.3 (0.5)
Rating of Baby’s father’s opinion about infant feeding (Important =1, Otherwise =0)			0.7 (0.4)	0.6 (0.5)	0.6 (0.5)
Concerns about family members’ opinion on infant feeding (cared =1, otherwise =0)			1.4 (0.3)	1.2 (0.4)	1.2 (0.4)
Are there cultural beliefs/ traditions in your place of origin about methods of feeding your baby? (Yes = 1, No = 0)			0.5 (0.5)	0.5 (0.6)	0.5 (0.6)
Received health care during pregnancy? (yes = 1, No =0)				13.9* (1.2)	20.2* (1.3)
Received healthcare from? (Nurse =1, Medical doctor/clinical officer =0)				1.9 (1.2)	2.3* (0.4)
Infant feeding attitude (score on Iowa scale)					1.1* (< 0.1)
Functional Social Support (score on scale)					1.1* (< 0.1)
Perceived Stress (score on Cohen’s scale)					0.9* (< 0.1)
*Model summary*					
(−2 Log Likelihood)	20.2	543.0	532.5	490.5	485.2
Chi-square statistics (*X*^*2*^)	339.4*	312.3*	231.6*	229.2*	243.3*
Observations analysed, n (%)	373 (54.1)				
Accuracy (%)	76.8	75.3	73.1	74.6	75.1

***p < 0.001, **p < 0.01, *p < 0.05

### Determinants of EFF relative to MF

Five variables in Model 5 were statistically significant determinants of EFF relative to MF at *p* < 0.05 (Table 5): the national guideline of EFF, the existence of cultural beliefs/traditions about infant feeding in the woman’s place of origin, pregnancy related healthcare received while living with HIV, received pregnancy healthcare from a nurse or midwife, and perceived stress.

Firstly, for a Black mother whose national guideline is EFF (1), compared to those whose guideline is EBF while on ART (0), had greater relative risk of choosing EFF over MF, holding all other variables in the model constant. Black mothers living with HIV and residing in Ottawa or Miami (relative to Port Harcourt) were several times more likely to practice EFF than MF.

Secondly, among mothers who said “yes” to the existence of cultural beliefs and traditions about infant feeding methods in their place of origin versus those who said “no,” the relative risk of practicing EFF relative to MF declined by a factor of 0.2, holding all other variables in the model constant. That is, mothers were less likely to practice EFF and more likely to practice MF if they reported cultural beliefs and practices about methods of feeding their babies in their places of origin. Thirdly, for mothers who received pregnancy related healthcare while living with HIV compared to those who did not, the relative risk of practicing EFF relative to MF increased by a factor of 21.2, holding all other factors in the model constant. Mothers who received healthcare during pregnancy were 21.2 times more likely to practice EFF instead of MF. Furthermore, among Black mothers living with HIV who received healthcare from a nurse or midwife instead of a medical doctor or clinical officer, the relative risk of EFF relative to MF increased by a factor of 3.1. Mothers who received pregnancy healthcare via a nurse or midwife were more likely to practice EFF instead of MF than those who received healthcare from a medical doctor or clinical officer. Finally, for a unit increase in a mother’s perceived stress score, the relative risk of EFF relative to MF was reduced by a factor of 0.9. Mothers with increased perceived stress were less likely to practice EFF and more likely to practice MF.

### Determinants of EBF relative to MF

Five variables were statistically significant determinants of EBF relative to MF in Model 5 (Table 6): healthcare received during pregnancy, healthcare received from a nurse or midwife, IIFAS, functional social support score, and Cohen’s PSS.

First, if a Black mother living with HIV received healthcare during pregnancy, the relative risk for her preferring EBF over MF would be expected to increase by a factor of 20.2, given that other variables in the model were held constant. In a broader view, mothers who received healthcare were more likely to practice EBF instead of MF. Second, for mothers who received the healthcare from a nurse or midwife compared to a medical doctor or clinical officer, the relative risk of EBF relative to MF increased by a factor of 2.3. Third, if a mother increased her IIFAS score by one unit, the relative risk for her choosing EBF over MF would be expected to increase by a factor of 1.1, given that other variables in Model 5 were held constant. Next, if the mother’s functional social support score increased by one unit, then the relative risk of her preferring EBF to MF would increase by a factor of 1.1. Lastly, a unit increase in the mother’s Cohen’s PSS would decrease the relative risk of her preferring EBF to MF by a factor of 0.9. In a general sense, an increase in Cohen’s PSS reduced the mother’s likelihood of choosing EBF over MF. Conversely, the mothers were more likely to prefer MF to EBF when they presented higher perceived stress scores.

## Discussion

This section discusses the statistically significant determinants of infant feeding practices in the study samples. These include the EFF guideline, cultural beliefs and practices, healthcare system, healthcare personnel, infant feeding attitudes, social support, and perceived stress. Key findings discussed were: a national guideline of EFF associated with an increased likelihood of EFF over MF; cultural beliefs and practices about infant feeding associated with a decreased likelihood of EFF over MF; receiving health care during pregnancy by HIV+ women was associated with an increased likelihood of EFF over MF, and increased EBF over MF; receiving the health care through a nurse or a midwife was associated with an increased likelihood of EFF over MF, and increased EBF over MF; infant feeding attitude score was associated with an increased likelihood of EFF over MF, and increased EBF over MF; and finally, functional social support score was associated with an increased likelihood of EFF over MF, and increased EBF over MF.

### National guideline

The national guideline of EFF for mothers living with HIV in Canada and the US was associated with increased likelihood of choosing EFF over MF. One principal reason for this would likely be deference by the mothers in Canada and the US to healthcare professional’s advice. This is perhaps because mothers might see the advice of health professionals concerning EFF as being the best practice, aligning with approved national guidelines in Canada and the US. In these national contexts, fear of being incriminated for not adhering to national guidelines may be an important factor for explaining why many women living with HIV in Ottawa and Miami choose EFF over other infant feeding methods. Howbeit, statistics in Table 1 show that a few mothers were not adhering to the guideline in their countries. For example, in Ottawa 9.2% (MF =3.4% and EBF = 5.8%) and in Miami 12.5% (MF =7.3% and EBF = 5.2%) were not adhering to EFF. Also, in Port Harcourt, 33.3% (MF = 15.2% and EFF = 18.1%) were not adhering to the guideline of EBF. Although many of the mothers have their reasons for their practices, non-adherence to guideline recommendations on infant feeding by mothers living with HIV is a critical factor in MTCT of HIV, including maternal and child health. A clear understanding of the guideline over the cultural and perceived Westernized infant feeding norms may be a vital pathway in addressing HIV spread, maternal, and child health. Many women who newly migrated to Canada perceived formula feeding as the modern, Westernized infant feeding method [39]. This perspective may appear realistic in historical statistics. For example, before 1965, breastfeeding initiation among Canadian mothers was less than 25% [40]. Only 17% of mothers in Canada in 2003 and 26% in 2011/2012 breastfed exclusively for 6 months or more [41]. These statistics imply that most Canadian women were formula feeding their babies. Over 70% of mothers in Ottawa each year, between 2011 and 2015, reported introducing formula before their infants had reached 6 months of age [41]. EBF in the first 6 months of infant life was as low as 13.6% among Canadian women, demonstrating their inclination toward formula feeding [42]. This perception can also explain why many Black women living with HIV prefer EFF over EBF. In the US, although 79% of mothers-initiated breastfeeding in 2011, only 18.8% practised EBF up to 6 months, and 26.7% were breastfeeding at 12 months [43]. The guideline of EFF compared to EBF while on ART increased the relative risk of EFF over MF. The preceding result implied that mothers residing in Canada or the US where the national guideline is EFF were more likely to practice EFF over MF than mothers in Nigeria. In contrast, our analysis showed that (EBF) exclusive breastfeeding relative to mixed feeding (MF) was not significantly determined by the Nigerian national guidelines of exclusive breastfeeding (EBF) while on ART. This may be due to family pressure [44] and prevalent cultural norms of MF, in which water and supplementary foods are administered in addition to breastmilk. The other highly probable alternative for Nigerian women would be EBF, which, in economic terms, would be the most feasible; and, again, for women living with HIV, it is the recommended option for guarding against MTCT when the mother is taking ART and has an undetectable HIV viral load. This is especially feasible when mothers have clearly understood the minimal risks of MTCT under these conditions.

### Cultural beliefs and practices

Descriptive analysis show that 21.5% of mothers from all sites (Table 2) affirmed that there are cultural beliefs and practices in their places of origin about how to feed their babies. Again 18.3% of the women perceived that cultural beliefs and practices influenced their infant feeding practices at a rating of “much” or “very much”. The inferential analysis (Tables 5 and 6) shows that the beliefs and practices concerning methods of infant feeding in the mothers’ cultures of origin decrease the mothers’ likelihood of choosing EFF over MF. This finding may be corroborating the perception of the few mothers (18.3%) who perceived that their infant feeding practices is much or very much influenced by cultural beliefs and practices. In line with the finding, a traditional norm whereby Black African women experience social pressure to MF has been documented in a systematic review [45]. It is customary in many parts of sub-Saharan Africa for mothers to feed their babies breastmilk, water, tea, porridge, and the like within the babies’ first few weeks of life [1, 46, 47]. Other studies in the South-South region of Nigeria, where Port Harcourt is located, have found that mothers experience pressures from family members to follow the traditional rites of giving their babies water, herbs, and teas alongside breastfeeding [48]. MF is a cultural practice – a way of treating colic, dehydration, and gastrointestinal distress [10, 49]. Where cultural expectations about infant feeding methods are not in line with national implementation of WHO guidelines, Black women face a lot of tension in their practices of infant feeding method. Such tension is accentuated by HIV-related stigma. For instance, when they choose formula feeding over breastfeeding in adhering to guidelines, they may experience stigma from their community for not acting in the culturally accepted way of mothering [50, 51]. If they choose to breastfeed, they may also experience stigma for attempting to infect the baby with HIV, particularly where the family or community knows about her HIV status [27, 51, 52]. Although HIV stigma is not a fitting variable in the model used in this paper, it is a very important factor that interacts with the mother’s cultural expectations to complicate her practices of infant feeding practices.

### Healthcare system

This study found that mothers who received healthcare during their pregnancies while living with HIV were more likely to practice EFF over MF than were mothers who did not receive such healthcare. Similarly, mothers who received healthcare during their pregnancy while also receiving ART were more likely to chose EBF over MF. This result is corroborated by findings that the health system supports the WHO/national recommendations on infant feeding practices [45]. Three platforms through which the health system provides infant feeding support include pre-delivery maternal counseling, post-delivery maternal support, and counseling from health workers [22, 53–55]. While healthcare systems can be nurturers or enablers of approved recommendations, the extant literature has shown that healthcare workers can have divergent influences on mothers’ infant feeding practices. Healthcare workers are likely to give adapted messages based on what they believed to be the best feeding practices for mothers [49, 56, 57]. For example, some studies have found that EFF has been suggested for women of high socioeconomic status while EBF has been recommended to mothers of low socioeconomic status [58, 59]. In other instances, health workers have been concerned that EBF would be challenging for women they perceived as undernourished, and, in some cases, healthcare workers have not supported six-month EBF because they believed the practice was unfeasible for women who would be leaving their infants at home with caregivers when they resumed work in early postpartum [58]. Mixed messaging was reported when women living with HIV were informed that breastfeeding is a mode of MTCT, while EBF is a means of prevention, without a clear explanation about the exact risk of transmission through breastfeeding with and without ART [60].

### Healthcare personnel

Mothers who received pregnancy healthcare from a nurse or midwife instead of a medical doctor or clinical officer were more likely to practice EFF compared to MF. They were also more likely to practice EBF than MF. Although a systematic review found that both trained doctors and nurses drive positive change on breastfeeding [61], some others show that medical doctors, including family physicians, do not receive sufficient training about supporting breastfeeding [62–64]. This, perhaps, represents a need for focused training on the details of infant feeding recommendations, including EFF and EBF for mothers living with HIV. Many physicians do not advocate breastfeeding for fear that it may culminate in parental guilt among some mothers [64], but this is not the case if parents are adequately educated about their practices [65]. While medical doctors provide counseling, nurses have an educational role in breastfeeding advocacy; they are responsible for providing educational supports to women, so mothers can make informed decisions to formula feed and administer the formula in a safe and nurturing way [66].

### Mothers’ infant feeding attitudes

This research has found that a unit increase in a mother’s IIFAS score predicted her choosing EBF over MF at a statistically significant level. This result is congruent with two past research findings which showed that IIFAS score was associated with infant feeding practices and breastfeeding duration among breastfeeding women [67, 68]. A study in rural Western Australia found that women with more positive attitudes towards breastfeeding had longer durations of EBF [69, 70]. Another study found that women who perceived breastfeeding to be healthier, easier, and more convenient breastfeed longer than those who perceive it to be restrictive, inconvenient and uncomfortable [71]. Thus, attitude towards breastfeeding, as measured by the IIFAS, is a predictor of EBF, as found in this study.

### Functional social support

Functional social support was associated with an increased likelihood of EBF over MF. This may be linked with the fact that functional social support provides emotional, information and material need of the mother to help her fully to adhere to the guideline of EBF. Many mothers without such support may lack information about ART, or not fully understand the reduction in the risk of MTCT of HIV when they are on ART. Mothers residing in many parts of Africa with little access to safe and nutritionally adequate alternatives to breastmilk can only allay fears of MTCT and practice EBF with sufficient functional social support. These levels of social support become a motivation for adhering to ART and help them understand the safety of EBF when their viral loads are undetectable. In 2016, WHO recommended being fully supported for ART adherence, for mothers living with HIV in countries where EBF is encouraged [6]. Hence, both ART and EBF can be fostered by functional social support as indicated in our finding. A lifelong ART, including adherence counselling and promotion of EBF and its duration would be key elements of social support system for mothers living with HIV. In line with the present findings, some recent studies have found that EBF is positively influenced by social support [72–74]. Beyond this, a study concludes that providing social support to mothers, as well as to other family members (fathers and grandmothers) would improve infant feeding practices [74]. An earlier study also found that social support and material assistance are associated with a decreased likelihood of EBF cessation among mothers living with HIV in Dares Salaam, Tanzania [75].

### Perceived stress

Increased perceived stress has statistically significant, negative effects on both EFF relative to MF, and EBF relative to MF. Although direct, comparable research findings are sparse, several studies, to some degree, support this result. Another study found an association between parenting stress and a composite measure of caretaking difficulties, including infant feeding [76]. Similarly, it has been inferred that high levels of stress are associated with negative parenting practices [77], which would include infant feeding practices. Similarly, maternal stress has been negatively associated with a broad range of parenting skills, which would, again, include feeding practices [78]. A study linked lack of association between MF and perceived stress to insufficient variabilities in the outcome variables (breastfeeding initiation and duration) [79].

Many researchers have often analyzed the relationship between depression (an outcome of stress) and arrays of maternal parenting attributes (including infant feeding practices). The results of such analyses implicitly capture the effects of stress on maternal practices. A study has established that depressive symptoms early in the postpartum period may lower breastfeeding prevalence [80]. In a systematic review, it was inferred that mothers suffering from depression are less likely to initiate breastfeeding; if they do, it will be with decreased duration, and EBF will be less likely [81]. Based on a literature review, it was noted that negative outcomes for infants are due to maternal depression and disorganized infant feeding practices [82], which result in overfeeding or under-nutrition [34, 83, 84]. Another recent study has found that postpartum anxiety increases breastfeeding difficulties and may negatively affect breastfeeding behaviors [85].

#### Limitation

One limitation of the study was the difficulty we encountered to reach Black mothers living with HIV. This impacted on the study samples sizes, especially in Ottawa. Hence, participants were recruited through venue-based approach at times and places where they would normally convene as opposed to probability sampling. Although this may have limited the generalisation of our results, this approach has been developed, refined, and successfully used in several studies done by members of our research networks. Venue-based approach is an adaptation of the standard approach used in HIV research and provides knowledge of places or groups to target for interventions. To reduce the likelihood of bias in the venue-based sampling, recruitment was strategically spread to as many venues and meetings as possible.

##### Strength

The study provides an understanding of the roles of national guideline, professional advice from healthcare system, and social support in shaping the infant feeding practices of Black mothers living with HIV. It also, paints a clear picture of how cultural values that contradict the infant feeding guideline, and maternal stress can alter infant feeding practices. The paper also draws it strength from the diverse views of Black mothers of various backgrounds including North American born Black mothers, immigrant Black African mothers, and mothers still in their origin in Africa.

## Conclusions

Although the women in our study indicated that cultural expectations were a significant source of stress, the national guidelines on how to feed their infant and health providers advice had more significant impact on their decisions about infant feeding practices among mothers in Canada and US. The providers advice was congruent with WHO and national guidelines for infant feeding among mothers living, which is exclusive formula feeding (EFF). Some mothers in these two North American sites preferred to use mixed feeding, which was against the WHO/national policy recommendations, but they did not succumb to the pressures of cultural expectations from peers, family, and community members. Mothers from the Nigerian site, who were already taking ART, were also more compliant with the healthcare system advice of EBF over MF. It was interesting to see the influence of health care providers on these mothers’ infant feeding practices in all three sites: Canada, the US and Nigeria. This is especially so if the mothers have received healthcare through a nurse practitioner and have social support from friends and families. This speaks to the need for health care providers to have updated knowledge of advances in HIV prevention and care cascade. Women dealt with stress with other strategies such as hypervigilance, use of mixed methods feeding, etc.

These results point to the importance of health care providers providing evidence-based HIV prevention and care. Educational programs for health care providers such as nurses and midwives need to have HIV care as a core aspect of curriculum content. In addition, health care providers need to be practice in culturally safe and responsive manner to meet the needs of Black women who are living with HIV. Global and national guidelines also need to take into consideration the unique needs of those that are impacted by such policies. For example, Black women living with HIV should be meaningfully engaged with other stakeholders at the arenas where the WHO and national guidelines are developed to ensure better alignment with their socio-cultural contexts. Respective national governments are to provide continued training programs for healthcare workers concerning the rationale for the infant feeding policy recommendations and should educate medical doctors and clinicians to develop social support skills so that they may further teach mothers. Finally, given that some mothers especially those that have been historically excluded from decision making arenas (i.e. Black mothers) may not have the confidence to assert themselves in terms of seeking information to inform health care decisions, it is important to include family members in educational program. Public health education must actively engage families and communities in enlightenment campaigns and community workshops about appropriate infant feeding practices for mothers living with HIV. This will help to them to understand their roles in providing social support to mothers and reduce the pressure to meet cultural expectations of motherhood. It may also help reduce the stigma associated with not breastfeeding. This education will help community members to understand that there are many other reasons outside HIV that could prevent a mother from breastfeeding. Healthcare providers and workers are well-positioned to educate other community and social service providers who will, in turn, educate mothers and their social networks on infant feeding recommendations. Routine visits to healthcare centers for counseling about infant feeding and medication adherence should be undertaken by the mothers themselves, in line with the advice of healthcare workers.

## Data Availability

Data analysis activities are currently underway. To request access to study data, please contact JE, the principal investigator for the project.

## Abbreviations

HIV: Human Immunodeficiency Virus
ART: Antiretroviral Therapy
EFF: Exclusive formula feeding
EBF: Exclusive breastfeeding while on ART and viral load monitoring
MF: Mixed feeding
RR: Relative risk
WHO: World Health Organisations, Statistical Package for Social Sciences
MTCT: Mother to Child transmission
US: United States of America
ACB: African, Caribbean, and Black
AIDS: Acquired Immunodeficiency Syndrome
IIFAS: Iowa Infant Feeding Attitudes Scale
PSS: Perceived Stress Scale
